# Supplementary material for: Analysis of PPARγ Signaling Activity in Psoriasis
Source: Int J Mol Sci. 2021 Aug 10;22(16):8603. doi: 10.3390/ijms22168603 (PMC8395241; doi:10.3390/ijms22168603)
Supplement: Supplementary file 1 [file ijms-22-08603-s001.zip › Supplemental materials_Analysis of PPARg signaling activity in psoriasis/Pathway models/Models images and html files/PPARG inhibits phenotypes assotiated with psoriasis/241980.html]

contact hypersensitivity


# Disease contact hypersensitivity

|  |  |
| --- | --- |
| URN | urn:agi-meshdis:Hypersensitivity%2c%20Contact |
| Total Entities | 0 |
| Connectivity | 1114 |
| Name | contact hypersensitivity |

---

|  |  |
| --- | --- |
| Pathway | PPARG inhibits phenotypes assotiated with psoriasis |
|  | efects selected |

---

|  |  |
| --- | --- |
| MedScan ID | 9006053 |

---

|  |  |
| --- | --- |
| Alias | Contact Hypersensitivity |
|  | contact allergy |
|  | contact phenomena |
|  | retest contact hypersensitivity |
|  | contact phenomenons |
|  | contact phenomenon |
|  | contact hyper-sensitivity |
|  | Contact Hypersensitivities |
|  | contactual destruction phenomenon |
|  | contact allergies |

---

|  |  |
| --- | --- |
| MeSH ID | M0006053 |

---

|  |  |
| --- | --- |
| MeSH Heading | Hypersensitivity, Contact |

---
